# A Meta-Analysis of the Effect of Bacillus Calmette-Guérin Vaccination Against Bovine Tuberculosis: Is Perfect the Enemy of Good?

**DOI:** 10.3389/fvets.2021.637580

**Published:** 2021-02-18

**Authors:** Sreenidhi Srinivasan, Andrew J. K. Conlan, Laurel A. Easterling, Christian Herrera, Premanshu Dandapat, Maroudam Veerasami, Gobena Ameni, Naresh Jindal, Gopal Dhinakar Raj, James Wood, Nick Juleff, Douwe Bakker, Martin Vordermeier, Vivek Kapur

**Affiliations:** ^1^Department of Animal Science, The Pennsylvania State University, University Park, PA, United States; ^2^The Huck Institutes of the Life Sciences, The Pennsylvania State University, University Park, PA, United States; ^3^Disease Dynamics Unit, Department of Veterinary Medicine, University of Cambridge, Cambridge, United Kingdom; ^4^School of Veterinary Medicine, University of Pennsylvania, Philadelphia, PA, United States; ^5^Indian Veterinary Research Institute, Eastern Regional Station, Kolkata, India; ^6^Cisgen Biotech Discoveries Pvt Ltd, Chennai, India; ^7^Aklilu Lemma Institute of Pathobiology, Addis Ababa University, Addis Ababa, Ethiopia; ^8^Department of Veterinary Public Health and Epidemiology, Lala Lajpat Rai University of Veterinary and Animal Sciences, Hisar, India; ^9^Translational Research Platform for Veterinary Biological, Tamil Nadu University of Veterinary and Animal Sciences, Chennai, India; ^10^The Bill & Melinda Gates Foundation, Seattle, WA, United States; ^11^Technical Consultant and Independent Researcher, Lelystad, Netherlands; ^12^Animal and Plant Health Agency, Addlestone, United Kingdom; ^13^Centre for Bovine Tuberculosis, Institute for Biological, Environmental and Rural Sciences, University of Aberystwyth, Aberystwyth, United Kingdom

**Keywords:** BCG vaccine, bovine tuberculosis, efficacy, cattle, control program

## Abstract

More than 50 million cattle are likely exposed to bovine tuberculosis (bTB) worldwide, highlighting an urgent need for bTB control strategies in low- and middle-income countries (LMICs) and other regions where the disease remains endemic and test-and-slaughter approaches are unfeasible. While Bacillus Calmette-Guérin (BCG) was first developed as a vaccine for use in cattle even before its widespread use in humans, its efficacy against bTB remains poorly understood. To address this important knowledge gap, we conducted a systematic review and meta-analysis to determine the direct efficacy of BCG against bTB challenge in cattle, and performed scenario analyses with transmission dynamic models incorporating direct and indirect vaccinal effects (“herd-immunity”) to assess potential impact on herd level disease control. The analysis shows a relative risk of infection of 0.75 (95% CI: 0.68, 0.82) in 1,902 vaccinates as compared with 1,667 controls, corresponding to a direct vaccine efficacy of 25% (95% CI: 18, 32). Importantly, scenario analyses considering both direct and indirect effects suggest that disease prevalence could be driven down close to Officially TB-Free (OTF) status (<0.1%), if BCG were introduced in the next 10-year time period in low to moderate (<15%) prevalence settings, and that 50–95% of cumulative cases may be averted over the next 50 years even in high (20–40%) disease burden settings with immediate implementation of BCG vaccination. Taken together, the analyses suggest that BCG vaccination may help accelerate control of bTB in endemic settings, particularly with early implementation in the face of dairy intensification in regions that currently lack effective bTB control programs.

## Introduction

Bovine tuberculosis (bTB) is a chronic infectious disease of cattle that is predominantly caused by *Mycobacterium bovis*, a zoonotic agent (1). The disease remains endemic in most low- and middle-income countries (LMICs) where it negatively impacts livestock productivity and represents a significant threat to public health. A live attenuated strain of *M. bovis*, the “Bacille de Calmette et Guérin” (BCG), has been used for experimental vaccination of cattle against bTB since 1913, well before its first trials in humans (2). Following Calmette and Guérin's promising early reports demonstrating safety of BCG and BCG-induced protection of cattle against experimental challenge with *M. bovis*, several trials were carried out in different countries in the early 20th century to better define its efficacy (3). Despite the repeated demonstration of BCG vaccine-induced protection in cattle, field use was not pursued because of the incomplete protection reported and, more importantly, because as a live attenuated vaccine, BCG sensitizes animals to the current World Organisation for Animal Health (OIE)-recommended purified protein derivatives (PPD)-based skin tests. This compromises the specificity of the standard tuberculin skin test and results in an inability to differentiate infected from vaccinated animals (DIVA) (4). Therefore, bTB control programs that use the OIE-prescribed tuberculin skin test also prohibit the use of BCG vaccination (5).

In the last decade, research in the field has focused on identifying antigens that are present in *M. bovis* and absent or not immunogenic in BCG. In particular, the antigens ESAT-6, CFP10 and Rv3615c have shown promise for differential diagnosis of bTB (4, 6, 7). The DIVA capability of these antigens has been demonstrated in both experimental and naturally infected animals, hence enabling the use of BCG vaccination as part of future bTB control programs (8, 9).

In order to assess the potential utility of BCG vaccination as a component of future bTB control programs, accurate estimates of its efficacy are first required. We sought to address this major knowledge gap through a systematic review and meta-analysis of the existing literature on efficacy of BCG vaccination against bTB in cattle. Vaccines can protect populations through two main modes of action—reducing the susceptibility of vaccinates to infection or by reducing the potential for transmission by vaccinates after infection. The latter effect is particularly relevant for BCG vaccination where reduction in pathology has been reported more frequently (10, 11) than sterilizing immunity. However, also estimating reduction in infectiousness requires either large scale field trials or carefully designed natural transmission studies (11). The vast majority of published efficacy studies of BCG in cattle have used experimental challenge models with a relatively high infectious dose that can only measure a reduction in susceptibility rather than assess the impact on transmission.

For our quantitative meta-analysis, we therefore focus on the effect of the vaccine to reduce susceptibility to infection (vaccine efficacy, ε_s_) defined by the presence or absence of visible lesions and/or confirmed by culture. We also review the evidence that supports what the possible range of efficacy BCG may offer in terms of a reduction in infectiousness (ε_I_) and explore the implications for disease control using a conceptual dynamic transmission model.

## Results

### Characteristics of Included Studies

**Figure 1:**
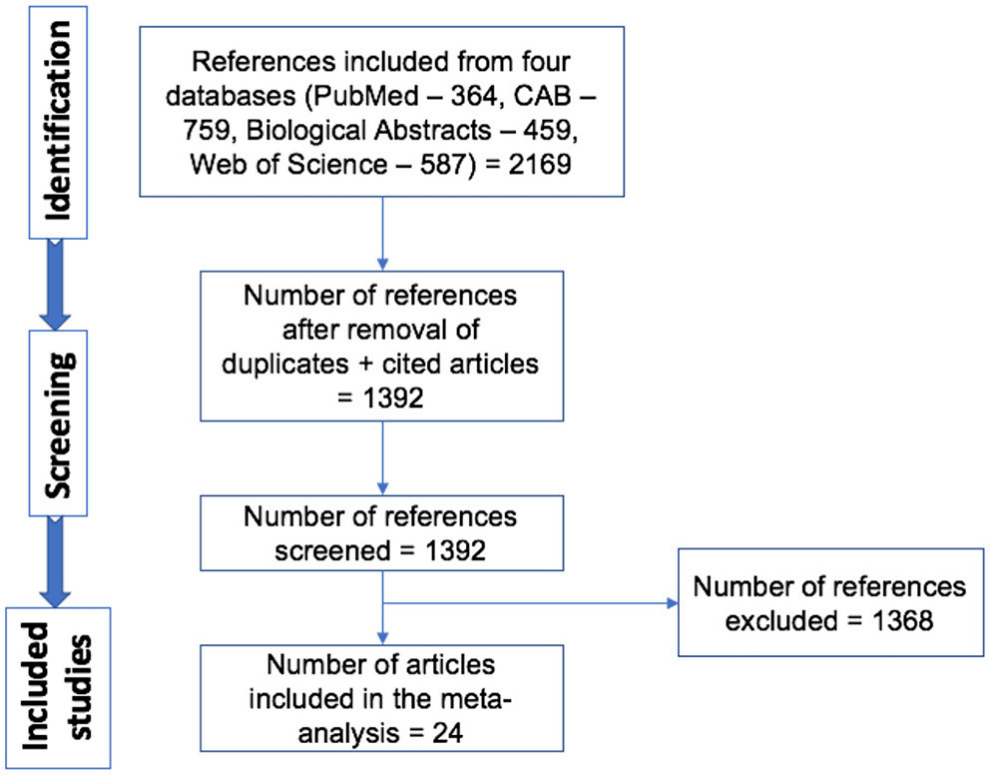
Schematic representation of literature selection procedure for the systematic review of BCG efficacy against bTB.

A total of 1,392 articles were screened, and 24 articles were included in the analyses (Figure 1) (12–35). In the instance that an article evaluated different doses and strains of BCG, different routes of vaccine administration, breeds of cattle, etc., each was considered as separate strata level data and included as a unique study. For instance, the study by Wedlock et al. (20) was extracted into three strata level data, representing the three different strains or variants of BCG tested (BCG Danish 1331, BCG Danish 1331 freeze-dried and BCG Pasteur 1173P2) (20). In total, 49 strata level data were extracted from the 24 publications included in the systematic review. The included publications spanned the time period from 1972 to 2018, and represented a total of 1,902 vaccinates compared against 1,667 control animals. All included studies are summarized in Table 1.

**Table 1:** List of strata-level data (*n* = 49) extracted from a total of 24 publications for inclusion in our meta-analysis.

**Database #**	**Authors**	**Source**	**Location**	**Tpos**	**Tneg**	**Cpos**	**Cneg**	**BCG dose**	**Route**	**Method**	**Infection measurement method**	**Sample allocation method**
101	Wedlock et al. (20)_1	BCG Pasteur 1173P2	New Zealand	9	1	10	0	1–4 × 10^6^ CFU	Subcutaneous	Experimental	Culture	Stratified Random Sample
101	Wedlock et al. (20)_2	BCG Danish 1331	New Zealand	8	2	10	0	1–4 × 10^6^ CFU	Subcutaneous	Experimental	Culture	Stratified Random Sample
101	Wedlock et al. (20)_3	BCG Danish freeze-dried	New Zealand	9	1	10	0	1–4 × 10^6^ CFU	Subcutaneous	Experimental	Culture	Stratified Random Sample
120	Buddle et al. (17)	BCG Pasteur 1173P2	New Zealand	16	2	7	2	1 × 10^5^ CFU	Subcutaneous	Experimental	Culture	Stratified Random Sample
579	Ameni et al. (35)	BCG Danish 1331	Ethiopia	14	9	22	4	1–4 × 10^6^ CFU	Subcutaneous	Natural	Culture	Random Sample
802	Buddle et al. (16)	BCG Pasteur 1173P2	New Zealand	5	4	5	4	5 × 10^5^ CFU	Subcutaneous	Experimental	Culture	Random Sample
826	Buddle et al. (14)_1	BCG Pasteur 1173P2	New Zealand	5	11	10	5	6 × 10^4^ CFU	Subcutaneous	Experimental	Culture	Not specified
826	Buddle et al. (14)_2	BCG Pasteur 1173P2	New Zealand	4	12	10	5	6 × 10^6^ CFU	Subcutaneous	Experimental	Culture	Not specified
828	Buddle et al. (15)_1	BCG Pasteur 1173P2	New Zealand	4	5	6	3	2 × 10^5^ CFU	Subcutaneous	Experimental	Culture	Random Sample
828	Buddle et al. (15)_2	BCG Pasteur 1173P2	New Zealand	4	5	6	3	2 × 10^3^ CFU	Subcutaneous	Experimental	Culture	Random Sample
828	Buddle et al. (15)_3	BCG Pasteur 1173P2	New Zealand	3	6	6	3	2 × 10^5^ CFU	Intratracheal	Experimental	Culture	Random Sample
956	Berggren (13)	BCG Glaxo	Malawi	75	129	82	128	8–26 × 10^6^ CFU	Subcutaneous	Natural	Culture	Alternate calves vaccinated
1065	Wedlock et al. (22)	BCG Pasteur 1173P2	New Zealand	5	5	7	3	1 × 10^6^ CFU	Subcutaneous	Experimental	PM	Stratified Random Sample
1080	Buddle et al. (21)_1	BCG Pasteur 1173P2	New Zealand	3	7	7	3	1 × 10^6^ CFU	Subcutaneous	Experimental	PM	Stratified Random Sample
1080	Buddle et al. (21)_2	BCG Pasteur 1173P2	New Zealand	2	8	7	3	1 × 10^9^ CFU	Oral	Experimental	PM	Stratified Random Sample
1080	Buddle et al. (21)_3	BCG Pasteur 1173P2	New Zealand	3	7	7	3	*	Subcutaneous and oral	Experimental	PM	Stratified Random Sample
1213	Buddle et al. (18)_1	BCG Pasteur 1173P2	New Zealand	6	4	10	0	1 × 10^6^ CFU	Subcutaneous	Experimental	Culture	Random Sample
1213	Buddle et al. (18)_2	BCG Pasteur 1173P2	New Zealand	9	1	10	0	1 × 10^6^ CFU	Subcutaneous	Experimental	Culture	Random Sample
1213	Buddle et al. (18)_3	BCG Pasteur 1173P2	New Zealand	8	2	10	0	1 × 10^6^ CFU	Subcutaneous	Experimental	Culture	Random Sample
1263	De Klerk et al. (24)	BCG Pasteur 1173P2	South Africa	8	6	9	4	3.2 × 10^7^ CFU	Intramuscular	Combined	Culture	Random Sample
1266	Ameni et al. (23)	BCG Danish 1331	Ethiopia	4	9	11	3	1 × 10^6^ CFU	Subcutaneous	Natural	Culture	Random Sample
1296	Thom et al. (28)_1	BCG Danish 1331	UK	6	3	8	1	1–4 × 10^6^ CFU	Subcutaneous	Experimental	Culture	Stratified Random Sample
1296	Thom et al. (28)_2	BCG Danish 1331	UK	9	0	9	0	1–4 × 10^6^ CFU	Subcutaneous	Experimental	Culture	Stratified Random Sample
1303	Parlane et al. (31)_1	BCG Danish 1331	New Zealand	14	2	16	1	2–8 × 10^6^ CFU	Subcutaneous	Experimental	PM	Stratified Random Sample
1303	Parlane et al. (31)_2	BCG Danish 1331	New Zealand	11	4	16	1	2–8 × 10^6^ CFU	Subcutaneous	Experimental	PM	Stratified Random Sample
1304	Buddle et al. (29)_1	BCG Danish 1331	New Zealand	2	7	10	0	1–4 × 10^5^ CFU	Subcutaneous	Experimental	PM	Stratified Random Sample
1304	Buddle et al. (29)_2	BCG Danish 1331	New Zealand	3	6	10	0	1–4 × 10^6^ CFU	Subcutaneous	Experimental	PM	Stratified Random Sample
1337	Buddle et al. (33)	BCG Danish 1331	New Zealand	6	6	7	5	1.5 × 10^6^ CFU	Subcutaneous	Experimental	PM	Stratified Random Sample
1358	Nugent et al. (34)_1	BCG Danish 1331	New Zealand	0	30	8	122	1 × 10^8^ CFU	Oral	Natural	PM	Random Sample
1358	Nugent et al. (34)_2	BCG Danish 1331	New Zealand	1	33	8	122	3 × 10^5^ CFU	Subcutaneous	Natural	PM	Random Sample
1358	Nugent et al. (34)_3	BCG Danish 1331	New Zealand	3	169	9	108	1 × 10^8^ CFU	Oral	Natural	PM	Random Sample
1358	Nugent et al. (34)_4	BCG Danish 1331	New Zealand	11	166	12	74	1 × 10^8^ CFU	Oral	Natural	PM	Random Sample
1358	Nugent et al. (34)_5	BCG Danish 1331	New Zealand	12	156	26	83	1 × 10^8^ CFU	Oral	Natural	PM	Random Sample
1358	Nugent et al. (34)_6	BCG Danish 1331	New Zealand	4	93	8	81	1 × 10^8^ CFU	Oral	Natural	PM	Random Sample
1366	Buddle et al. (19)_1	BCG Pasteur 1173P2	New Zealand	6	4	9	1	1 × 10^8^ CFU	Oral	Experimental	PM	Stratified Random Sample
1366	Buddle et al. (19)_2	BCG Pasteur 1173P2	New Zealand	7	3	9	1	1 × 10^9^ CFU	Oral	Experimental	PM	Stratified Random Sample
1366	Buddle et al. (19)_3	BCG Pasteur 1173P2	New Zealand	5	5	9	1	1 × 10^6^ CFU	Subcutaneous	Experimental	PM	Stratified Random Sample
1371	Hope et al. (27)_1	BCG Danish 1331	UK	7	0	7	0	2 × 10^6^ CFU	Subcutaneous	Experimental	PM and Culture	Not specified
1371	Hope et al. (27)_2	BCG Pasteur 1173P2	UK	3	4	7	0	2 × 10^6^ CFU	Subcutaneous	Experimental	PM and Culture	Not specified
1373	Lopez-Valencia et al. (25)	BCG Tokyo	Mexico	6	59	15	51	1 × 10^6^ CFU	Subcutaneous	Natural	Skin test and IFNg assay	Alternate calves vaccinated
1379	Buddle et al. (26)_1	BCG Danish 1331	New Zealand	2	7	6	4	1 × 10^6^ CFU	Subcutaneous	Experimental	PM	Stratified Random Sample
1379	Buddle et al. (26)_2	BCG Danish 1331	New Zealand	5	4	6	4	1 × 10^8^ CFU	Oral	Experimental	PM	Stratified Random Sample
1379	Buddle et al. (26)_3	BCG Danish 1331	New Zealand	5	4	6	4	1 × 10^7^ CFU	Oral	Experimental	PM	Stratified Random Sample
1379	Buddle et al. (26)_4	BCG Danish 1331	New Zealand	6	3	6	4	1 × 10^6^ CFU	Oral	Experimental	PM	Stratified Random Sample
1383	Dean et al. (30)	BCG Danish 1331	UK	4	6	9	1	1 × 10^6^ CFU	Subcutaneous	Experimental	Culture	Not specified
1385	Dean et al. (32)_1	BCG Danish 1331	UK	8	2	8	1	1 × 10^6^ CFU	Subcutaneous	Experimental	Culture	Random Sample
1385	Dean et al. (32)_2	BCG Danish 1331	UK	5	5	8	1	5 × 10^5^ CFU	Subcutaneous and endobronchial	Experimental	Culture	Random Sample
1385	Dean et al. (32)_3	BCG Danish 1331	UK	5	5	8	1	1 × 10^6^ CFU	Endobronchial	Experimental	Culture	Random Sample
1410	Nugent et al. (36)	BCG Danish 1331	New Zealand	2	518	8	289	3 × 10^5^ CFU	Subcutaneous	Natural	PM and Culture	Block Randomization

* *Buddle et al. (21)_3 used a combination of Oral and Subcutaneous BCG; Study no. 1263 (24) was performed in buffaloes. Tpos and Cpos are animals classified as positive for bTB in vaccinates and controls, respectively. Tneg and Cneg are animals that remained negative for bTB in vaccinates and controls, respectively*.

*Multiple strata level data extracted from a single study are represented with numbers at the end in the Author column. For example, Database #101, Wedlock et al had 3 strata level data represented as Wedlock et al._1, Wedlock et al._2 and Wedlock et al._3. Buddle_a is Reference (14) and Buddle_b is Reference (15). a1, a2, and b1, b2 are strata level data of those respective studies*.

### Meta-Analysis

**Figure 2:**
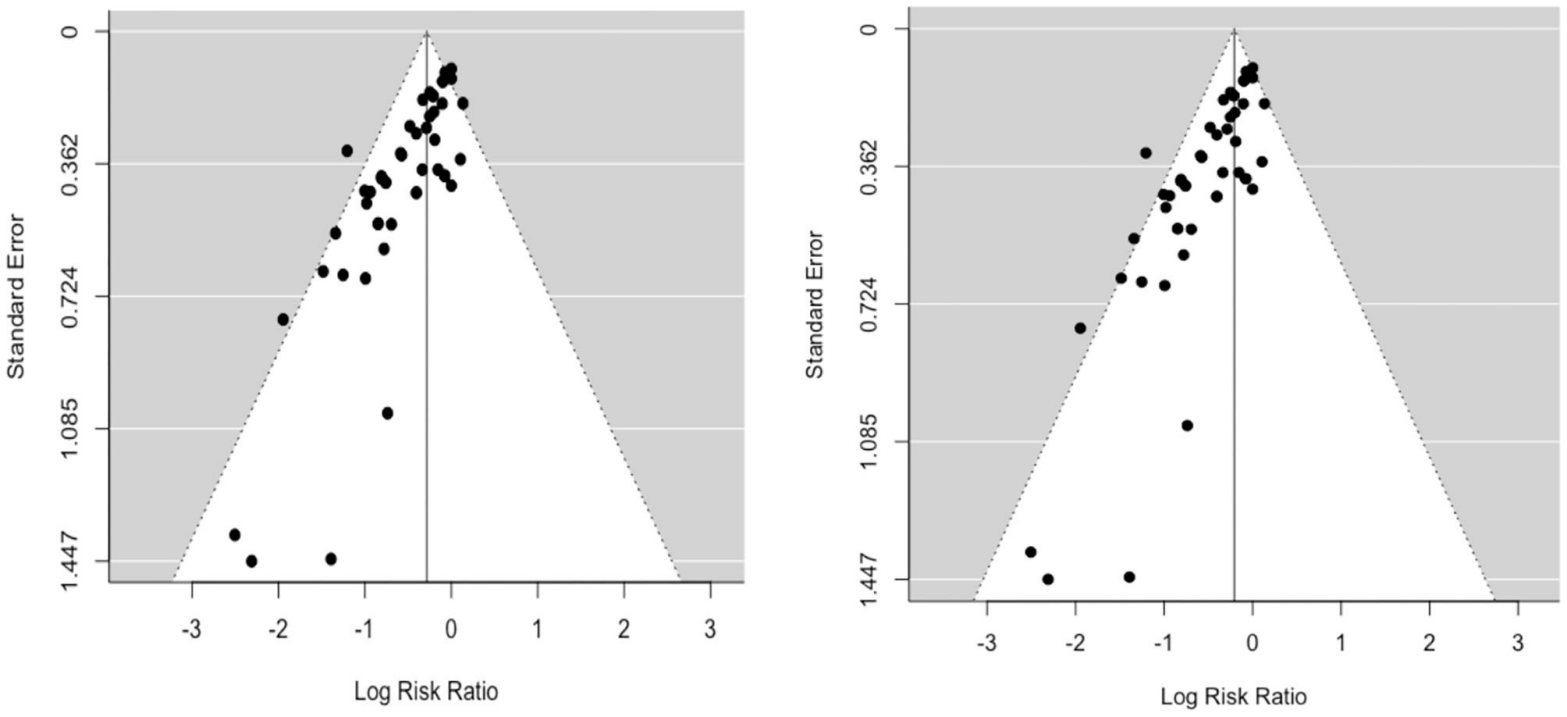
Funnel plots (left: RE; right: FE) of the log risk ratio against standard error demonstrate potential publication bias.

A funnel plot of the log risk ratio against standard error was constructed to assess potential publication bias (Figure 2). This revealed a large degree of asymmetry suggesting the presence of publication bias. There was no obvious difference in symmetry between random-effects (RE) and fixed-effects (FE) funnel plots. Similarly, visual inspection of the predicted vs. empirical observations (Normal Q-Q plot) also did not show any major differences in data fit for RE and FE models (Supplementary Figure 1).

**Figure 3:**
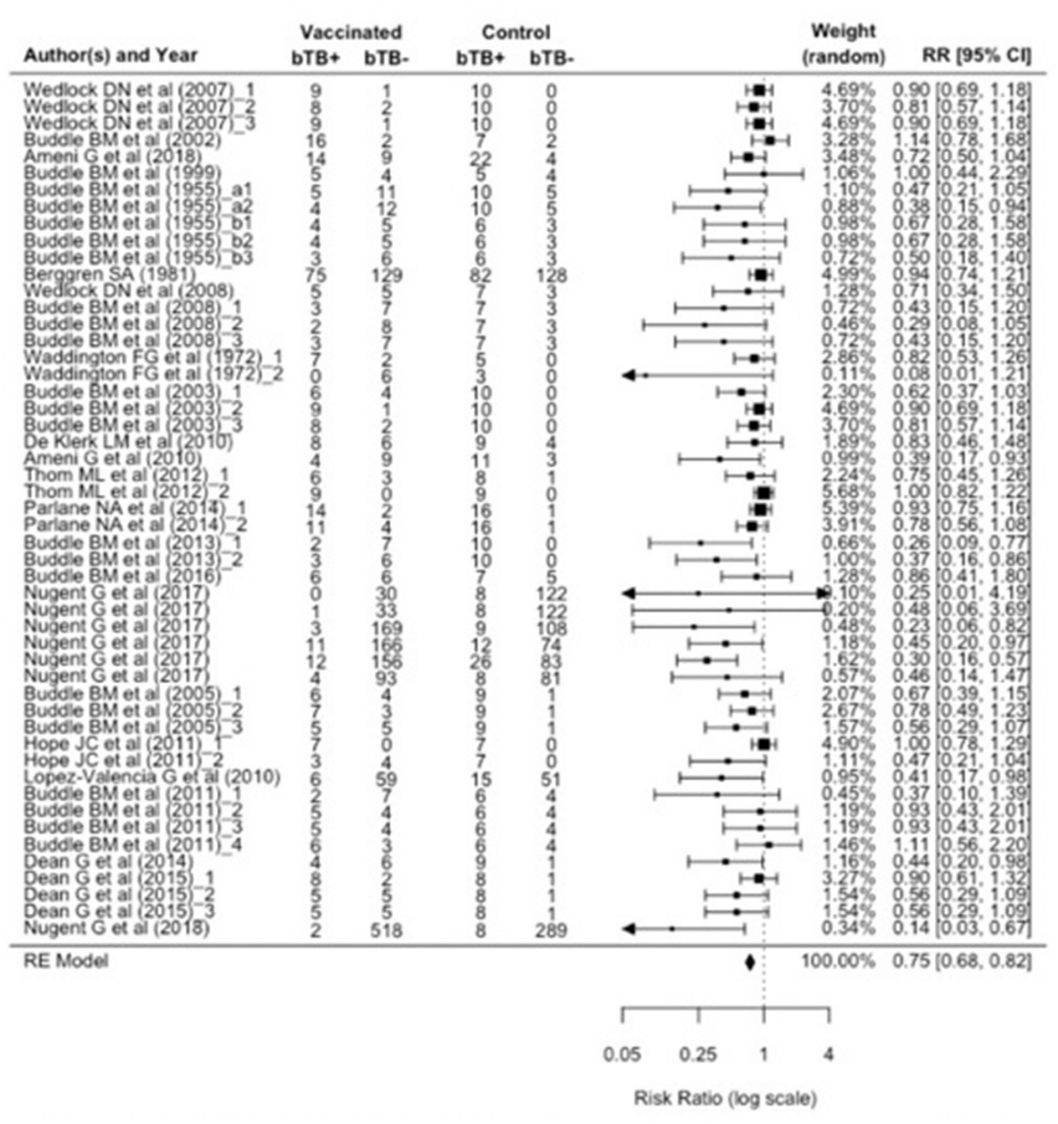
Forest plot visualizing the relative risk ratio calculated for each included publication in the meta-analysis. The weight given to each included publication per the RE model is shown. “bTB+” refers to the number of animals reported to have infection and/or pathology (per culture and/or postmortem examination). And “bTB-” refers to the number of animals not reported to have infection and/or pathology.

Given the suggestion of publication bias in the data, we focus on the RE model, which adjusts for variability between individual studies, as the more appropriate model to assess the relative risk ratio (RR). The relative risk ratio is defined as the probability of an outcome in an exposed group to that in an unexposed group (37). The RE model estimated RR to be 0.75 (95% CI: 0.68, 0.82), suggesting a 25% reduction in risk of infection (vaccine efficacy, ε_*S*_) as measured by PM and/or culture in BCG vaccinates compared to control animals. Cochran's (*Q*) value (*Q* = 76.1, *df* = 50, and *p* = 0.01) and Higgins statistic (*I*^2^ = 32.1%) were computed to test for heterogeneity (*I* ^2^ < 50% represents low heterogeneity). While both the *Q* value and *I*^2^ statistic are classical measures of heterogeneity, neither is comprehensive since Cochran's (*Q*) suffers low power and *I*^2^ is imprecise in the case of meta-analyses of relatively small numbers of included studies. We used a forest plot to graphically summarize the variation in RR between studies (Figure 3).

**Figure 4:**
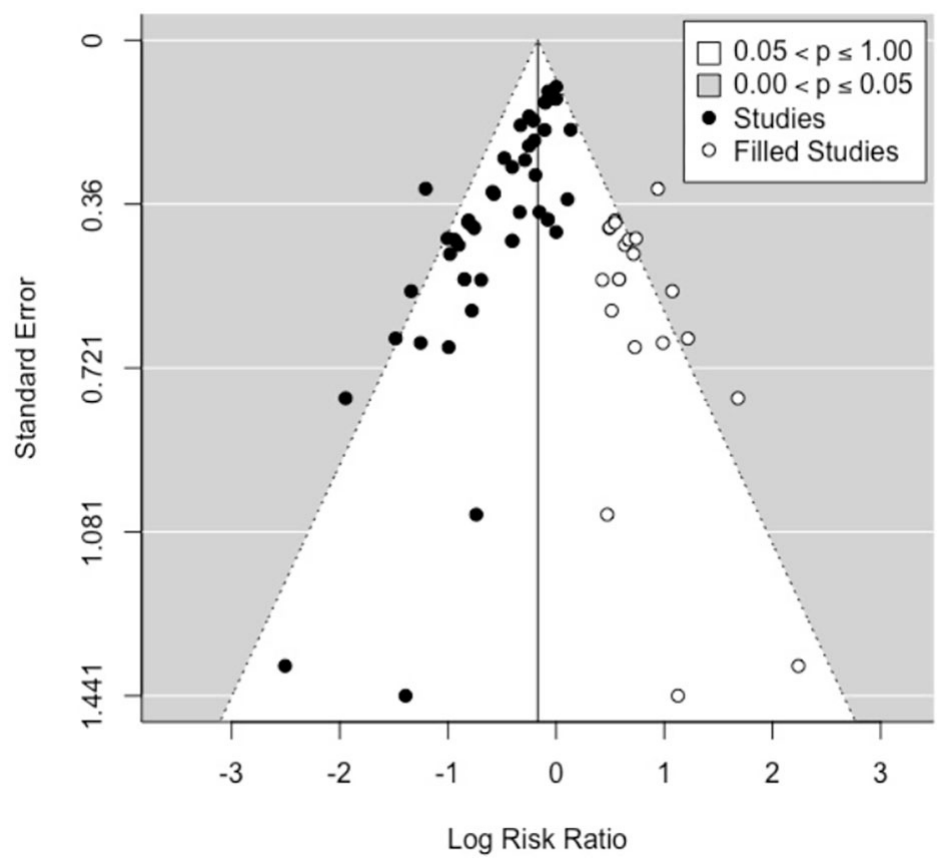
Funnel plot showing the 21 studies (open circles, on the right side) imputed by the trim-and-fill method to carry out a sensitivity analysis for publication bias.

To explore the potential impact of publication bias on the estimated RR, we carried out a sensitivity analysis using the trim and fill method (38). This is an algorithmic method to adjust for publication bias in a meta-analysis by imputing the values of missing studies (39). Here, the estimated number of missing studies on the right side of the funnel plot was found to be 21 (Figure 4). The Paul-Mantel method was used due to convergence issues with the maximum likelihood based methods (Maximum-Likelihood (ML), Restricted Maximum-Likelihood (40) and Empirical Bayes (EB) estimators). The adjusted RR estimate per this sensitivity analysis was found to be 0.84 (95% CI: 0.73, 0.98).

### Meta-Regression

Several important biological factors varied between published studies, which *a priori* could have an impact on the estimated vaccine efficacy. Such confounding factors could also potentially lead to systematic patterns of bias as shown by the funnel plot analysis. We therefore constructed a multivariable meta-regression model using these factors to explore whether they could improve model fit and to assess the relative effect of these biological variables on estimates of vaccine efficacy. The factors included BCG source, dose, route, whether or not revaccination was performed, and challenge method (Table 2).

**Table 2:** Multivariable meta-regression of the selected predictors on BCG efficacy (R^2^ = 49.10%, *n* = 49).

**Moderators**	**Categories**	**RR (95% CI)**	***p*-value (RE)**
Revaccination	No	Reference	
	Yes	1.04 (0.79, 1.38)	0.77
BCG route	Endobronchial	Reference	
	Intramuscular	2.16 (0.55, 8.50)	0.27
	Intratracheal	1.01 (0.28, 3.67)	0.99
	Oral	2.04 (0.75, 5.53)	0.16
	Subcutaneous	1.46 (0.71, 3.00)	0.3
	Subcutaneous and Endobronchial	1 (0.38, 2.66)	1.00
Challenge method	Natural	Reference	
	Experimental	0.83 (0.40, 1.70)	0.61
BCG source	Danish 1331	Reference	
	Danish freeze-dried	1.14 (0.77, 1.68)	0.51
	Glaxo	12.14 (1.52, 97.09)	0.02
	Pasteur 1173P2	0.98 (0.77, 1.23)	0.84
BCG Dose		0.68 (0.31, 1.51)	0.35
Time from vaccination to challenge		1.00 (0.99, 1.001)	0.5
Length of exposure to challenge		0.998 (0.997, 1)	0.06

An omnibus test of all the moderator variables (QM = 23.7, df = 13, *p* = 0.03) indicates that the explained variance by the model is greater than the unexplained variance. However, this amounts to only 49.1% of the total heterogeneity and only one moderator (BCG Glaxo source) has a RR significantly different than 1 (at the 95% level). Given the relatively small sample sizes, reflective of the logistical constraints of experimental studies, this lack of statistical significance is unsurprising and does not rule out the potential biological importance of these variables and highlights the critical need for additional well-powered investigations to better assess the impact of these confounders on overall vaccine efficacy.

### Implications for bTB Control

To explore the implications of the estimated efficacy of vaccination of ε_*S*_ ~ 25% for disease control, we carried out scenario analyses using a conceptual herd level transmission dynamic model. Given the potential density dependence of transmission rates of bTB within herds, intensification of production in emerging dairy industries is a particular concern for LMICs (41). Thus, we consider a scenario with an initial herd size of 30 but growing in size at a rate of 15% per year to 134 animals after 50 years. Motivated by estimates from India we consider an initial bTB prevalence (5, 10, and 15%) together with a set of higher prevalence scenarios (20, 30, and 40%) (42). There are currently no published quantitative estimates of transmission rates from LMICs, hence for this model population we use a density dependent transmission function estimated from herds in Great Britain (11).

**Figure 5:**
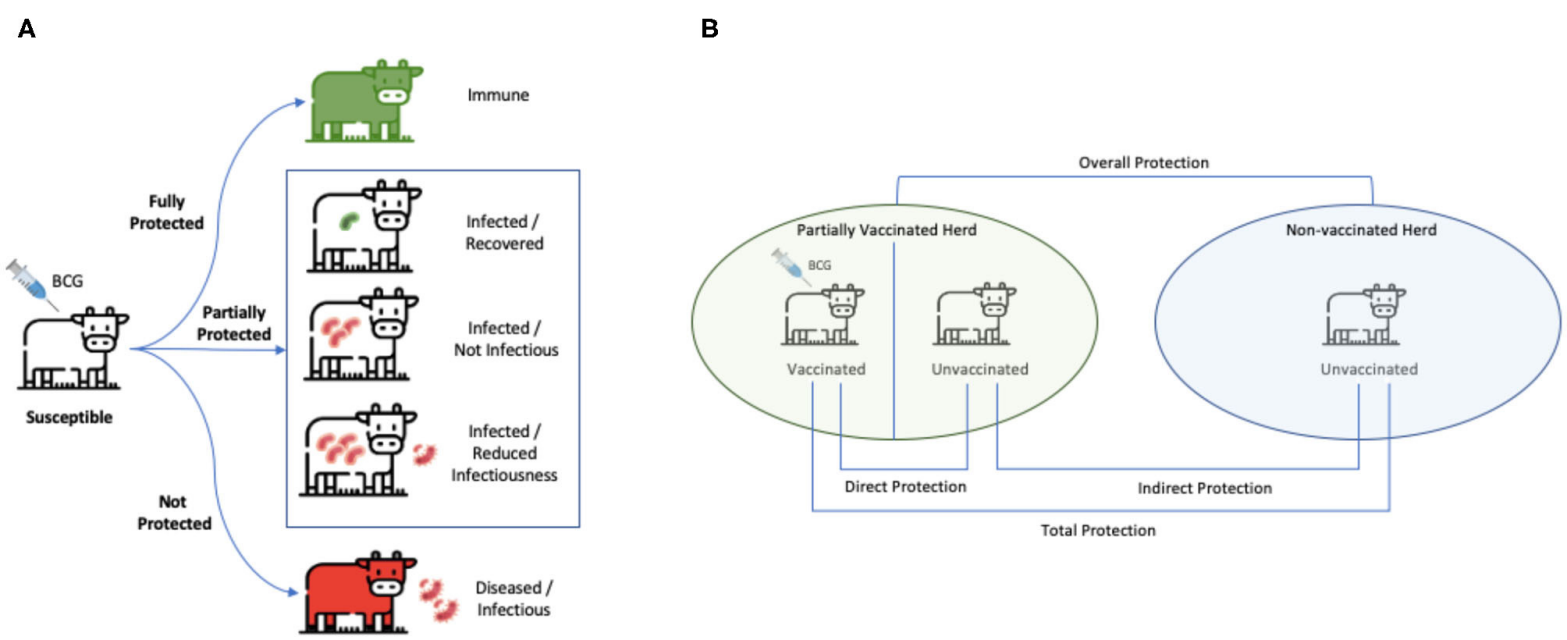
Measures of vaccine-induced protection. **(A)** BCG vaccine confers a spectrum of protection ranging from complete (for a limited time period in certain population) to none. While completely protected individuals are immune, and unprotected ones succumb to infection and become heavily diseased, BCG can also be partially protective by reducing risk of disease transmission to unvaccinated individuals. **(B)** The green herd is partially vaccinated and the blue herd is unvaccinated. “Direct protection” is the reduction in susceptibility of vaccinated animals (as measured by **ε**_**s**_). “Indirect protection” is a reduction in transmission due to any reduction in infectiousness of vaccinated individuals that become infected (as measured by **ε**_**I**_) in addition to the reduction in transmission from herd immunity effects. “Total” protection is the risk of infection in vaccinated animals compared to that of unvaccinated animals. “Overall protection” is the reduction in rate of transmission in a population with vaccination program compared to that in a population with no vaccination program. This stems from both vaccinated and unvaccinated individuals and is used to evaluate the impact of a vaccination program at the population level. **(B)** Is adapted from Halloran et al. (43).

In the absence of estimates of indirect BCG effects based on empirical trials, we used data from long-term natural challenge models to estimate the relative contribution of indirect effects when force of infection is low (representative of field setting). Overall efficacy in those recent field trials included in this meta-analysis [excluding the older Berggren (13) study] was estimated to be of 61% (95% CI: 40, 74) for natural transmission compared to 18% (95% CI: 11, 24) for experimental challenge studies (Table 3). The key distinction between experimental challenge and natural transmission studies—beyond being more representative of a field setting—is that the latter measures the total effect of vaccination (Figure 5). The total effect of vaccination will be greater than the direct protection of individuals (ε_*s*_) due to the reduction in transmission from herd immunity effects, along with any additional protection from reduction in infectiousness (ε_*I*_) of vaccinated individuals.

**Table 3:** Natural transmission studies included in the meta-analysis.

**Database #**	**Authors**	**Location**	**BCG source**	**BCG route**	**BCG dose**	**Controls_n**	**Vaccinates_n**	**Reported efficacy**
579	Ameni et al. (35)	Ethiopia	BCG Danish 1331	Subcutaneous	1–4 × 10^6^ CFU	26	23	30%
1266	Ameni et al. (23)	Ethiopia	BCG Danish 1331	Subcutaneous	1 × 10^6^ CFU	14	13	60%
1358	Nugent et al. (34)	New Zealand	BCG Danish 1331	Oral and Subcutaneous	1 × 10^8^ CFU	531	644	67%
1373	Lopez-Valencia et al. (25)	Mexico	BCG Tokyo	Subcutaneous	1 × 10^6^ CFU	66	65	60%
1410	Nugent et al. (36)	New Zealand	BCG Danish 1331	Subcutaneous	3 × 10^5^ CFU	297	520	85%

*The studies conducted in Ethiopia by Ameni et al. (23, 35) used a reactor to sentinel ratio of >1. Nugent et al. (34) included 1,286 free-ranging cattle (676 vaccinates and 531 naïve controls) stocked at low densities, and challenged with a low force of infection, mimicking a more representative field setting. This was followed up with a subsequent field study (36) conducted in ~800 free-ranging cattle that were followed up for over 3 years and similarly showed a relatively high overall protective efficacy of ~85%. Lopez-Valencia et al. (25) did not conduct necropsy*.

**Figure 6:**
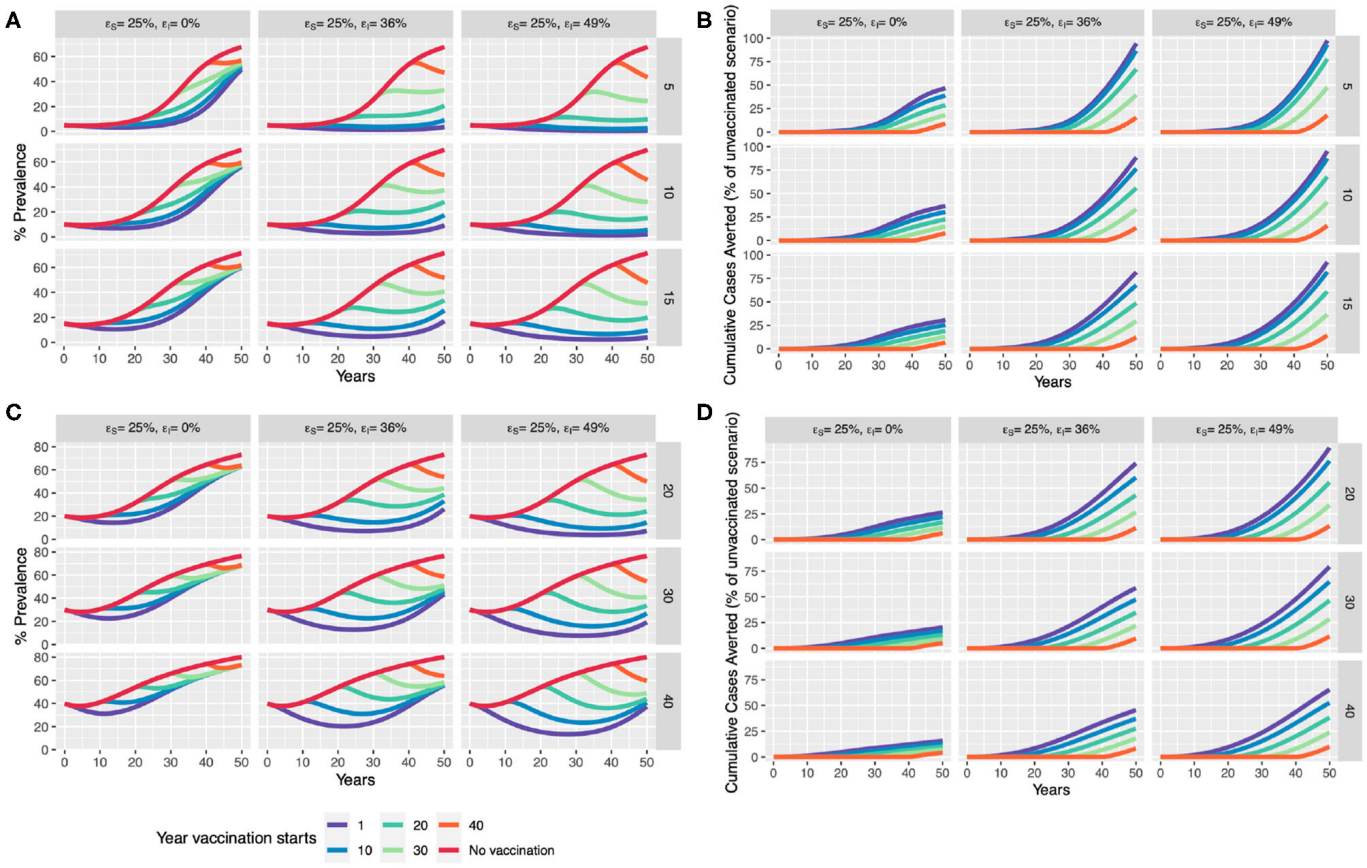
Scenario analysis for impact of BCG vaccination in an expanding dairy herd. We compare a baseline “control” scenario (with no vaccination) to the introduction of whole-herd vaccination at 1, 10, 20, 30, and 40 years which only provides direct protection (ε_s_ of 25%), and one with an additional reduction in infectiousness of vaccinates (ε_I_ of 36 and 49%). We compare the effect of vaccination in terms of % prevalence and cumulative cases averted with assumed initial **(A,B)** moderate prevalence of 5, 10, and 15% (rows), and **(C,D)** high prevalence of 20, 30, and 40%.

In the absence of control, the null model predicts a gradual increase in prevalence with herd size saturating around ~70% for all initial conditions (Figure 6). We considered a vaccine efficacy to reduce infectiousness (ε_I_) at two hypothetical thresholds: (i) ~36%, approximating the difference between average overall efficacy in the natural transmission studies (0.61) and the overall direct efficacy as revealed by the meta-analyses of 0.25; and (ii) ~49%—representing the difference between the upper bound of efficacy in the natural transmission studies (0.74) and overall direct efficacy as revealed by the meta-analyses of 0.25.

Our model scenarios illustrate the benefits of early intervention. Even with only a modest efficacy (ε_s_) of 25%, a window of up to 25 years of slightly lower than the current disease prevalence levels may be achieved (Figure 6A), should BCG be implemented immediately in the low to moderate prevalence scenario (5, 10, 15%). With an additional reduction in infectiousness (ε_I_) of vaccinates (49%), disease prevalence could be driven down close to Officially TB Free (OTF) status (<0.1% per the European Union) if BCG were introduced in the next 10-year time period in low to moderate prevalence settings. Immediate implementation, but with a lower ε_I_ of 36%, prevalence may start to rise again in 30–40-years, driven by increased herd size, suggesting that other strategies will also be required.

These scenarios predict considerable benefits in terms of reducing cumulative cases if BCG is implemented now. With an impact of vaccination on infectiousness, between ~80 and 100% of cases can be saved in 50 years, driven by the strength of indirect effects on transmission (Figure 6B). Encouragingly, this model also predicts that from ~50 to 95% of cumulative cases can be averted in higher disease burden settings (>15%) if acted upon now (Figure 6).

## Discussion

Following promising initial studies conducted by Calmette, Guérin and others that showed both safety of BCG and BCG-induced protection of cattle against both natural and experimental challenge with *M. bovis*, several trials were carried out through the early and mid 20th century in many different countries to better assess and define the efficacy of ancestral variants of BCG in cattle and showed varying levels of protection (3, 44) (Supplementary Table 1). While BCG went on to become the most widely used of all human vaccines (and the only available vaccine against human TB), it has not been considered for routine use in domestic livestock despite early promise. This is primarily because of the fact that BCG sensitizes animals to the widely used and OIE-recommended PPD based skin tests, which precludes its use where skin test-based control is being actively pursued (5), and it is with this context that an Expert Committee of the WHO/FAO Expert Committee on Zoonoses (45) stated that: “generally speaking, vaccination has no place in the eradication of tuberculosis in cattle.”

The early proof-of-concept experiments conducted by Calmette and Guérin in the early 1900s to demonstrate safety of BCG and protection against progressive TB in cattle were replicated and refined by several other investigators in both Europe and the Americas (44, 46–53). Their findings led to considerable international interest in exploring the possibility of eradicating bTB by vaccinating cattle with BCG as has been reviewed elsewhere (3, 10, 54–57). As part of the current systematic review, we extracted data from eight studies that were published between 1930 and 1972 (Supplementary Table 1). However, given that these studies used ancestral strains of BCG and doses that are not possible to accurately estimate, these studies were excluded from the formal analyses. However, it is noteworthy that these studies provided key foundational evidence that highlighted both the variable protection afforded by BCG in experimental and natural challenge, as well as showed that BCG vaccination sensitizes animals to tuberculins and hence would confound ongoing test and slaughter tuberculosis eradication programs. However, recent attempts to develop BCG-compatible diagnostic tests have shown great promise and paved the way for implementation of BCG-based control programs in LMICs and other endemic regions (7–9). Thus, there is renewed interest in exploring the use of BCG vaccination as a component of national control programs, particularly where test and slaughter is unfeasible (58, 59).

We systematically reviewed the literature to obtain estimates of the efficacy of BCG against bTB and assessed its potential utility in future control programs. The analyses suggest an overall vaccine efficacy of 25% (95% CI: 18, 32) as measured by the presence of visible lesions and/or culture. The potential contribution of vaccine strain as a confounder or predictor of BCG efficacy is intriguing. This is relevant since a majority of the studies that were included in the current review used either BCG Pasteur 1173P2 (9 out of 24 studies) or BCG Danish 1331 (10 out of 24 studies). The meta-regression model suggests that neither of these strains had any significant effect on the observed heterogeneity. However, given the small sample size, further investigations may be warranted to assess the robustness of these inferences.

Given the large variability in the age at which animals were vaccinated in the included studies, the ideal timing for vaccination could not be subjected to rigorous analysis. While there is evidence in the published literature showing that BCG vaccination within a few days of birth (<1 month of age) can induce high levels of protective immunity (18, 60), there is a need for more studies in relevant settings, such as in bTB-endemic regions where high burden of environmental mycobacteria has been reported (61). In these settings, some studies suggest prior sensitization with environmental mycobacteria prevent induction of protection in calves, while others report that exposure to *M. avium* helps induce protection against *M. bovis* (17, 62). It is plausible that these seemingly contradictory results could be because exposure to different environmental mycobacteria could either enhance or reduce BCG's efficacy, and hence, future studies are needed to help clarify the role of these confounders to vaccine efficacy. Curiously, despite the early recognition by Calmette and Guerin and other investigators of the need for revaccination (at annual or 18 month intervals) in order to maintain protection (3), only a total of five studies performed revaccination, highlighting the scarcity of reliable data available on duration of immunity and revaccination intervals to formulate evidence-based approaches to control bTB (13, 16, 18, 24, 31). Thus, it is important to assess the true duration of immunity and role, if any, of revaccination to obtain better estimates of the efficacy of BCG and inform the parameterization of robust transmission dynamics and econometric models that may be used to guide development and implementation of future cost-effective vaccine-based intervention strategies (18, 31).

The roles of dose and type of challenge (experimental challenge or natural infection) were also investigated. A total of 17 studies that are part of this meta-analysis performed experimental challenge of their animals to infection. These studies call for high dose infection protocols in order to generate high levels of infection in control animals, which do not reflect the reality in natural conditions where animals most likely are not exposed to such high numbers of virulent bacteria as a single bolus. Such experimental challenge models with high infection doses do not allow BCG protection to be revealed. One reason for this may be that these models are often used for the development of novel vaccines aimed at improving BCG efficacy and not *per se* to study BCG efficacy. This highlights the importance of conducting robust natural transmission studies in field conditions and in endemic settings where the real need for vaccination lies. It is noteworthy that most experimental trials have used *M. bovis* as the challenge strain. However, *M. bovis* is not the only causative agent of bTB since *M. tuberculosis* and other members of the *M. tuberculosis* complex are frequently isolated from cattle samples, and certain species of non-tuberculous mycobacteria (NTM) that contain virulence factors encoded in the Region of Difference-1 are also known to cause TB-like lesions (63, 64). Hence, future studies are needed to better estimate efficacy of BCG vaccine-induced protection with locally relevant bTB causing mycobacteria other than *M. bovis*.

It is important to note that there are several limitations to systematic reviews and meta-analyses. They can provide a summary of published evidence about a specific question, but our ability to answer it is limited by variation in experimental design and the consistency and completeness of reporting. This systematic review was limited to studies in English indexed in the four databases screened, and hence may have missed relevant investigations. Studies that did not report essential details were excluded. It is not surprising that systematic biases in the fit of the meta-analysis models suggest existence of publication bias and the potential to inflate or underestimate estimates of direct vaccine efficacy. One of the primary limitations of the included studies is that the reported measures of protection, evidence of lesions at necropsy and culture, together with immune reactions, are the current “gold standards,” but have variable sensitivity and specificity (65). In order to address any potential bias in measurement of the outcome, we focused on studies which reported culture or necropsy data for calculation of RR estimation and further analyses. An exception was made however for the Lopez-Valencia et al. (25) study since it was one of only five estimates from a recent natural transmission study. This study considered an animal to be infected if there were positive reactions to the tuberculin skin test and the IFNγ release assay upon stimulation with PPD-B and recombinant ESAT6-CFP10 (25). While many studies followed a simple or stratified sampling strategy for randomization, two studies (13, 25) used a more systematic approach of vaccinating every alternate calf in the herd which may have introduced potential biases from the randomization process (Table 1). However, a rigorous assessment of other risks of bias due to deviations from intended interventions, missing outcome data and selection of reported result could not be performed due to lack of access to the complete data or detailed experimental protocols from these studies. Another limitation is that only the direct effect of vaccination (reduction in susceptibility, ε_*S*_) could be estimated from the experimental challenge experiments (66). This is important, in particular, given our scenario analyses which demonstrate the transformative effect an additional reduction in infectiousness of vaccinates (ε_*I*_) could have (67, 68). Thus, studies to assess the impact of BCG on infectiousness of vaccinates through natural transmission experiments or field trials are urgently needed (67).

Herd-size is perhaps the most important risk factor associated with transmission of bTB (69), leading to the common assumption that transmission is density dependent and increases with herd size (70, 71). This poses a particular challenge for bTB control in emerging dairy markets in LMICs where intensification of the dairy sector may make achieving control increasingly difficult over time. We developed herd level transmission dynamic models and performed scenario analyses using deterministic models to illustrate this challenge. The results of these analyses are striking—and suggest that despite the relatively modest direct protection afforded by BCG, a strong case may be made for implementing vaccination for bTB control sooner rather than later, with overall benefits reducing progressively over time to intervention (72) (Figure 6). Overall, a leaky vaccine like BCG could still play a pivotal role in disease control, particularly if implemented sooner than later in the face of dairy intensification efforts in bTB endemic countries.

Finally, even while scenario analyses are for illustrative purposes, there are several limitations to these analyses that we recognize and should be addressed in future investigations. To begin with, in the absence of surveillance programs in LMICs, transmission estimates are lacking and should be determined to accurately assess risk. Given that recent studies show increasing prevalence in intensifying cattle herds in endemic settings (69), these observations have major implications for informing and implementing disease control policy and suggest a potential role for BCG vaccination that has hitherto remained unexplored. Next, in order to realize herd-level benefits of vaccination, estimating the reduction in the risk of transmission from vaccinated individuals (ε_*I*_) is crucial. Animal trials in relevant endemic settings are urgently needed to evaluate the potential of BCG to reduce onward transmission in the field (11).

It is important to note that in addition to cattle, tuberculosis affects a wide variety of livestock and both free-living and captive wildlife species including goats, sheep, pigs, cervids, wild boars, badgers, brushtail possums, and ferrets, many of which are recognized as potential reservoir hosts. Indeed, experimental and field trials to study the response to BCG vaccination of some these species suggests that, while vaccination confers incomplete protection, it's use in domestic and captive or free-living wildlife species should be seriously considered to reduce risk of cross-species spillover (73). Alternative approaches to control bTB, such as test-and-segregate and treatment with anti-mycobacterial agents including isoniazid have also been explored (74, 75). Treatment with isoniazid is uneconomical and not advised given the long duration of treatment needed and the need to withdraw milk together with reports that bacterial shedding may resume as soon as isoniazid is withdrawn (76). Moreover, use of a first line antimycobacterial agent in food animals is of major concern due to potential for contribution to the spread of drug resistant tuberculosis (77). Over the past decade, research in the field has also focused on improving TB vaccination using alternative approaches including the heterologous prime-boost strategy, introducing genetic modifications in BCG strains to increase immunogenicity, and also completely replacing BCG with attenuated *M. bovis* strains (78–82). Heat-inactivated *M. bovis* vaccines have also recently shown promise in wild boars, pigs, red deer, badgers, and goats (73, 83–88). However, despite significant efforts and promising results in preclinical studies, there is only limited evidence from clinical and field trials for significant gains in efficacy of these newer generation or modified BCG vaccines. Interestingly, both heat-inactivated and attenuated vaccines for another major mycobacterial disease, paratuberculosis (Johne's disease), have been licensed and tested extensively for use in cattle and small ruminants, but their use is limited in countries because of potential for interference with tuberculin testing and diagnostic tools currently used in bTB eradication and control programs (89, 90).

In conclusion, in the short-term, in endemic regions where test and slaughter approaches to bTB control have not been shown to be effective or, BCG vaccination alongside a DIVA diagnostic test appears to be the most promising option in the near future. However, it is important to continue to develop and assess next generation vaccines as well as complementary strategies of disease control alongside that of BCG. For instance, the assessment of routine testing and segregation of reactor animals, mandatory pasteurization of milk fed to calves and sold for human consumption, enhanced husbandry practices, such as segregation of reactor or likely infected animals, herd certification policies for recruitment of disease-free animals, slaughterhouse surveillance, regulating movement and trade of reactor animals, etc., in conjunction with or as alternatives to BCG need to be rigorously evaluated in different production settings and epidemiological contexts. Together, our studies highlight an urgent need as well to perform sensitivity analyses and build econometric frameworks to assess the cost-benefit impacts of implementing vaccine-based control strategies to establish the business case for (or against) implementation of BCG vaccination as a component of a national bovine TB control program.

## Conclusion

This systematic review and meta-analysis of the efficacy of BCG vaccination in cattle together with transmission dynamic model-based scenario analyses provides strong evidence for the consideration of implementation of BCG vaccine-based bTB control strategies, particularly in LMICs and other high burden settings. Despite a relatively small but positive protective effect, conservative transmission models suggest an important role for BCG in limiting spread of the disease and buying time for improvement of vaccine efficacy or the development of alternative approaches to disease control. Taken together with the predicted increase in prevalence associated with intensification of dairy production, our investigations suggest that BCG vaccination may indeed be simply good (enough) to accelerate control of bTB in endemic settings.

## Methods

### Literature Search Strategy

A systematic search was performed for published articles reporting the effect of BCG against bTB in cattle as of February 24, 2020. Various combinations of Boolean operators and MeSH terms common to known articles of interest were evaluated before the following search terms were finalized: (BCG AND ((“mycobacterium bovis” OR tuberculosis) AND (cows OR cattle OR bovine) AND (protect^*^ OR effica^*^ OR lesion^*^ OR immune^*^ OR vaccin^*^))). In order to minimize publication bias, search terms were kept uniform across the four databases (PubMed, CAB, Biological Abstracts, and Web of Science), which were selected for their inclusion of major and minor international journals. No limits were placed on publication date, and only published studies were considered. EndNote X8, a citation software program, was used to organize the articles generated by the four databases as well as remove duplicate publications. The study conforms to Preferred Reporting Items for Systematic Reviews and Meta-analyses (PRISMA) guidelines (91).

### Study Inclusion Criteria

Inclusion and exclusion criteria are detailed in Table 4. All included studies compared the potentially protective effects induced by a BCG variant against natural or experimental challenge with bTB in a vaccinated group of cattle and a control group of unvaccinated cattle. Studies with no control group, such as surveys evaluating the effectiveness of vaccination campaigns, were excluded. Additionally, articles that failed to report the BCG vaccine strain tested were excluded (summarized in Supplementary Table 1). While efforts were made to identify articles manually, all final included articles were represented in the formal database search. Primary studies were included when available. However, review articles were included when the primary article was inaccessible and adequate information on BCG strain, bTB challenge, and protection was detailed for vaccinate and control groups.

**Table 4:** Study inclusion/exclusion criteria.

**Inclusion**	**Exclusion**
Bovine TB	Wrong disease
Cattle or buffalo	Wrong species
Used BCG to vaccinate (and specified the strain used)	Wrong vaccine
Evaluated efficacy of BCG vaccination	Wrong type of study
English	Language limitation: not in English
Full text of publication obtanied	Full-text unavailable
	Other

### Data Extraction

A uniform data extraction form was developed based on information of interest from pre-identified articles and used by each of the extractors. Study identifiers included author, publication date, title, journal, study location, and study time period. Headings for study design included control and vaccinate group sizes; BCG strain (Danish 1331, Danish freeze-dried, Glaxo, Pasteur 1173P2, Tokyo); BCG dose; and BCG administration route (subcutaneous, endobronchial, intratracheal, intramuscular, oral); cattle age at time of vaccination; revaccination timing, frequency, and dose (if applicable); animal breed; and time from vaccination to challenge. Headings detailing bTB challenge methods included challenge type (natural or experimental) and duration (from challenge to slaughter or end of study period); as well as challenge strain, route (intratracheal, subcutaneous, oral, endobronchial) and dose if challenge type was experimental. Headings for protection included the infection measurement method (post-mortem, culture), number of vaccinates and controls affected, and the protection percentage reported. Vaccine efficacy was measured based on culture growth, and in the absence of culture data, presence or absence of visible lung or lymph node lesions in the control and treatment groups was used.

Prior to the formal review of all articles in the pool, data extractors (SS, LE, and CH) conducted a pilot run of 20 random articles in order to test inclusion/exclusion criteria and finalize the data extraction form. SS, LE, and CH independently performed the formal review of all articles, which comprised an initial screening of article titles and abstracts for inclusion or exclusion. Full-text review for data extraction from included studies was performed by SS and LE. When discrepancies arose in study inclusion/exclusion or extracted data between reviewers, studies were collectively revisited and discussed in order to reach a final decision.

Included studies used common protection measurement methods, primarily post-mortem examination (PM) and/or culture. In the case that a study used both PM and culture to measure protection, preference was given to culture (65); in the case that lung and lymph node lesions were reported, we considered positivity per lung lesions for effect measure. We only included studies that specified the challenge strain. A majority of the included publications performed experimental (17/24) challenge and did not revaccinate animals (18/24). A complete list of all included and excluded studies is publicly available at https://doi.org/10.26208/pykx-8s25.

### Statistical Analysis

All statistical analyses were performed in R (version 3.5.0) using RStudio (version 1.1.442). Full source codes used for the statistical analyses are publicly available with this publication at https://doi.org/10.26208/pykx-8s25. Random effects (RE) (4) and fixed effects (FE) meta-analysis models were estimated using the “rma” function of the “metaphor” package and analyzed using additional functions from the “meta” package (92, 93). Funnel plots were constructed to assess for systematic bias in the fitted models. Cochran's Q statistic was computed to test for unexplained heterogeneity, and Higgin's statistic helped describe the inconsistencies in studies' results (94, 95). Key parameters used to define protocols for BCG vaccination trials that were judged on biological grounds to be important were assessed as moderators in a multivariate meta-regression model, estimated using the rma function of the metafor package.

Due to the variability between reporting of pathology between different studies, vaccine efficacy was measured as a binary dichotomous variable based on the presence or absence of culture growth, or visible lesions in the control and treatment groups. Vaccine efficacy is most commonly measured using the relative risk or risk ratio (RR) (4), defined as the ratio of attack rate in the vaccinated and control groups. As well as being more easily interpretable than other measures (such as odds ratios and risk differences), RR can be used to define vaccine efficacy (1-RR) and was estimated here per the RE model. The 2 × 2 contingency table below provides a summary of included sample size (Table 5).

**Table 5:** A summary of all studies included in the meta-analysis.

		**Infection**	
		**Yes**	**No**
Vaccination	Yes	362	1,540
	No	530	1,137

*Out of a total of 1,902 BCG vaccinated animals, 356 were found to be positive (either by culture and/or presence of visible lesions) for bTB while 1,502 remained negative. Similarly, out of 1,667 control animals, 520 were infected while 1,104 animals neither had lesions nor any growth in culture*.

### Scenario Analysis

Thus far, no study on BCG efficacy has attempted to estimate the efficacy of BCG to reduce the infectiousness of vaccinates who subsequently become infected. In the absence of empirical data, the reduction in lesions in vaccinated animals estimated from natural transmission studies may be considered as a proxy for reduction in infectiousness. While how the extent of lesions directly relates to a reduction in infectiousness and thus transmission risk was not quantified, this significant reduction in lesion severity following BCG vaccination likely contributes to reduction in risk of transmission from vaccinates to susceptible cattle. Here, we conducted scenario analyses to explore the potential implications for disease control and the importance of estimating this neglected—but critical—aspect of BCG efficacy. We consider this proportionate reduction in lesions as a plausible upper bound for the total vaccine-induced protection.

For a well-mixed population, the critical vaccination threshold (V_c_) for elimination of a disease can be calculated from the basic reproduction number (R_0_) using the formula V_c_ = (1 – 1/*R*_0_)/*E*, where *E* is the relative (combined direct and indirect) efficacy of the vaccine (96). For domesticated livestock species in organized settings, routine vaccinations are often performed for all animals, hence we can reframe the critical threshold in terms of the critical efficacy E_c_ = (1 – 1/*R*_0_). Thus, with a direct vaccine efficacy of 25% (95% CI: 18, 32), BCG could be expected to successfully eliminate infection from fully vaccinated herds when the within-herd R_0_ is 1.32 (95% CI, 1.20–1.45) or lower.

A simple deterministic model for the within-herd transmission of bTB was used to explore the potential impact of BCG vaccination in an expanding dairy sector. The model assumes a well-mixed herd with animals stratified into four epidemiological compartments. In an unvaccinated population, animals are either susceptible (*S*) or infected and potentially infectious (*I*). Vaccinated individuals (*V*) have a reduced risk of becoming infected and we add a final infected after vaccination compartment to model a reduced infectiousness for these animals (*I*_*V*_). Density dependence in transmission is modeled using the non-linear relationship estimated from herds in Great Britain (GB) using the so-called SOR model (71). The SOR model subdivides the infectious class into an occult (O) and reactive (R) groups that differ in their reaction to the tuberculin skin test, but also assumes infected animals are potentially infectious immediately and is thus structurally equivalent to the *SI*(*VI*_*V*_) model used here.

The full model equations are:

$$\begin{array}{l} \;\frac{dS}{dt}=\left(1-p\right)\mu N-\frac{\beta }{{\left(N/N_{m}\right)}^{q}}\left(I+\;\left(1-\varepsilon_{I}\right)\;Iv\right)\;S-\nu S\\ \;\frac{dI}{dt}=\frac{\beta }{{\left(N/N_{m}\right)}^{q}}\left(I+\;\left(1-\varepsilon_{\;I}\right)\;I_{V}\right)\;S-\nu I\\ \;\frac{dV}{dt}=p\mu N-\left(1-\varepsilon_{s}\right)\;\frac{\beta }{{\left(N/N_{m}\right)}^{q}}\left(I+\;\left(1-\varepsilon_{\;I}\right)\;I_{V}\right)\;V-\nu V\\ \frac{dI_{V}}{dt}=\left(1-\varepsilon_{s}\right)\;\frac{\beta }{{\left(N/N_{m}\right)}^{q}}\left(I+\;\left(1-\varepsilon_{\;I}\right)\;I_{V}\right)\;V-\nu I_{V} \end{array}$$

$$\begin{array}{l} \frac{dC}{dt}=\frac{\beta }{{\left(N/N_{m}\right)}^{q}}\left(I+\;\left(1-\varepsilon_{\;I}\right)\;I_{V}\right)S+\left(1-\varepsilon_{s}\right)\\ \;\frac{\beta }{{\left(N/N_{m}\right)}^{q}}\left(I+\;\left(1-\varepsilon_{I}\right)\;I_{V}\right)\;V\\ \frac{dN}{dt}=\left(\mu -\nu \right)N \end{array}$$

where ε_*s*_ is the efficacy of vaccination to protect against infection (reduction in susceptibility), ε_*I*_ is the efficacy of vaccination with respect to reducing the infectiousness of vaccinates that subsequently become infected and *N*_*m*_ is a constant centering parameter (=165) used for the estimation of q in the original GB model. β is a transmission parameter which we fix using the assumed initial prevalence (I_0_) and initial herd size (N_0_) for each scenario and q measures the strength of density dependence of transmission with q = 0 corresponding to density dependence and q = 1 frequency dependence. For the default scenario we use the point estimate from Conlan et al. (71) of q = 0.15 and present an alternative weaker density dependent scenario (q = 0.71, upper bound of approximate posterior distribution) in Supplementary Material.

For illustration we consider a herd with initial size N_0_ = 30, a 20% per annum replacement rate (ν = 0.2 per year) and per capita birth rate μ = 1.15 ν to give a population growth rate of 15% per year ($N=N_{0}e^{-\left(\mu -\upsilon \right)t}$).

The basic reproduction ratio for this model (for fixed herd size N_0_) can be calculated (next generation method) as:

$$\begin{array}{l} R_{0}=\;\frac{\beta }{\upsilon }N_{0}{\left(N_{0}/N_{m}\right)}^{-q} \end{array}$$

We set the (97) value of β for each scenario by assuming that the initial population is at the equilibrium point of the constant population model. Thus, at *t* = 0, we assume that:

$$\begin{array}{l} R_{0}=1/\left(1-I_{0}/N_{0}\right) \end{array}$$

and thus set:

$$\begin{array}{l} \beta =\;\frac{\nu R_{0}{\left(N_{0}/N_{m}\right)}^{q}}{N_{0}} \end{array}$$

Full source codes used for the statistical analyses are publicly available with this publication at https://doi.org/10.26208/pykx-8s25.

## Author Contributions

SS, LE, and VK designed the study. SS, LE, and CH performed data extraction. SS and AC compiled the final data and analyzed the data. SS drafted the paper. SS, AC, PD, MVe, GA, GR, JW, NJ, DB, MVo, and VK contributed to the writing. All authors contributed to the article and approved the submitted version.

## Conflict of Interest

MV is the Director of Cisgen Biotech Discoveries Pvt. Ltd., Chennai, India. The remaining authors declare that the research was conducted in the absence of any commercial or financial relationships that could be construed as a potential conflict of interest.
