# Corrigendum: A Meta-Analysis of the Effect of Bacillus Calmette-Guérin Vaccination Against Bovine Tuberculosis: Is Perfect the Enemy of Good?

**DOI:** 10.3389/fvets.2021.716565

**Published:** 2021-07-16

**Authors:** Sreenidhi Srinivasan, Andrew J. K. Conlan, Laurel A. Easterling, Christian Herrera, Premanshu Dandapat, Maroudam Veerasami, Gobena Ameni, Naresh Jindal, Gopal Dhinakar Raj, James Wood, Nick Juleff, Douwe Bakker, Martin Vordermeier, Vivek Kapur

**Affiliations:** ^1^Department of Animal Science, The Pennsylvania State University, University Park, PA, United States; ^2^The Huck Institutes of the Life Sciences, The Pennsylvania State University, University Park, PA, United States; ^3^Disease Dynamics Unit, Department of Veterinary Medicine, University of Cambridge, Cambridge, United Kingdom; ^4^School of Veterinary Medicine, University of Pennsylvania, Philadelphia, PA, United States; ^5^Indian Veterinary Research Institute, Eastern Regional Station, Kolkata, India; ^6^Cisgen Biotech Discoveries Pvt Ltd, Chennai, India; ^7^Aklilu Lemma Institute of Pathobiology, Addis Ababa University, Addis Ababa, Ethiopia; ^8^Department of Veterinary Public Health and Epidemiology, Lala Lajpat Rai University of Veterinary and Animal Sciences, Hisar, India; ^9^Translational Research Platform for Veterinary Biological, Tamil Nadu University of Veterinary and Animal Sciences, Chennai, India; ^10^The Bill & Melinda Gates Foundation, Seattle, WA, United States; ^11^Technical Consultant and Independent Researcher, Lelystad, Netherlands; ^12^Animal and Plant Health Agency, Addlestone, United Kingdom; ^13^Centre for Bovine Tuberculosis, Institute for Biological, Environmental and Rural Sciences, University of Aberystwyth, Aberystwyth, United Kingdom

**Keywords:** BCG vaccine, bovine tuberculosis, efficacy, cattle, control program

In the original article, there was an error. The source code link provided in Methods section is wrong in 3 occurrences. The correct link to the full source code is https://doi.org/10.26208/pykx-8s25.

A correction has been made to *Methods, Statistical Analysis, First Paragraph*:

Full source codes used for the statistical analyses are publicly available with this publication at https://doi.org/10.26208/pykx-8s25.

A correction has been made to *Methods, Scenario Analysis, Last Paragraph*:

Full source codes used for the statistical analyses are publicly available with this publication at https://doi.org/10.26208/pykx-8s25.

A correction has been made to *Methods, Data Extraction, Last Paragraph*:

A complete list of all included and excluded studies is publicly available at https://doi.org/10.26208/pykx-8s25.

The authors apologize for this error and state that this does not change the scientific conclusions of the article in any way. The original article has been updated.

