# Supplementary figures and images for: A Meta-Analysis of the Effect of Bacillus Calmette-Guérin Vaccination Against Bovine Tuberculosis: Is Perfect the Enemy of Good?
Source: Front Vet Sci. 2021 Feb 18;8:637580. doi: 10.3389/fvets.2021.637580 (PMC7930010; doi:10.3389/fvets.2021.637580)

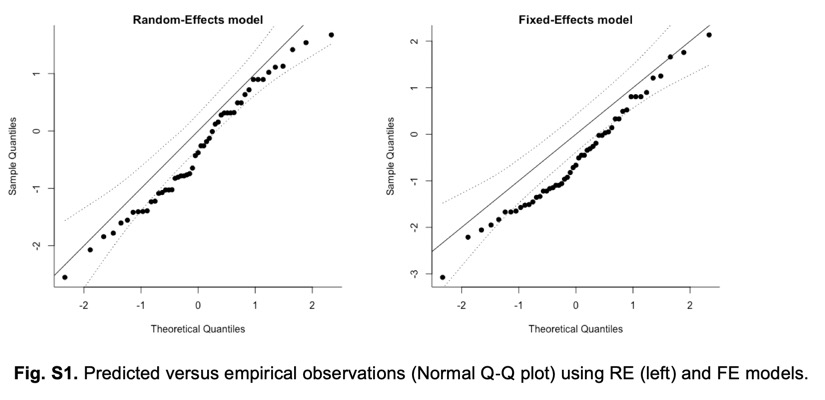

Supplement: Supplementary file 1 [file Image_1.jpg]
